# The Post-Operative Results in Nine Cases of Gastro-Enterostomy, with One Case in Which More Than Two-Thirds of the Stomach Was Resected for Cancer

**Published:** 1915-10

**Authors:** Gaston Torrance

**Affiliations:** Birmingham, Ala.; Surgeon to Birmingham Infirmary


### THE POST-OPERATIVE RESULTS IN NINE CASES OF GASTRO-ENTEROSTOMY, WITH ONE CASE IN WHICH MORE THAN TWO-THIRDS OF THE STOMACH WAS RESECTED FOR CANCER.

By Gaston Torrance, M. D., Birmingham, Ala.
Surgeon to Birmingham Infirmary.

The following cases have all been operated on within the past six years:

Case 1. Perforating gastric ulcer; closed by suture; no-loop gastro-enterostomy; operation six years ago.

This patient was seen about an hour after perforation, and gave the following history:

She had eaten a hearty meal eight hours before, and had been out for a walk of three or four miles, and came home feeling better than usual; she attempted to lift a suit case of books weighing about fifty pounds and immediately felt a soreness in the epigastrium; about ten minutes later, she felt a sudden, sharp pain, instead, in this region, and vomited, and had a severe rigor; no blood in vomitus.

The following is the history previous to perforation:

Mrs. K., age 29; says she has had digestive trouble since she was 12 years of age, and at times has had severe pain in the epigastrium; has always had ulcers in her mouth. Has three children; had some gastric trouble after the birth of the first child seven years ago, and has not been at all well since the birth of the last child, which is seven months old.

For the past three months, she has had more digestive trouble than ever before; large ulcers in her mouth; sour stomach and has taken soda to relieve it; there has been considerable gas distension; pain from one to three hours after eating and worse in the evening after dinner; no vomiting; there has been a continuous soreness, and she has been unable to wear her corset for two months; constipated; and has lost about ten pounds in weight.

---

(Read before the Chattahoochee Valley Medical and Surgical Association at West Point, Georgia, July 15, 1914).

When I first saw her the pulse was 96 and temperature normal; there was marked tenderness and pain over the epigastrium and considerable rigidity of the recti muscles.

I made a diagnosis of a perforation at or near the pylorus, and advised immediate operation.

The white blood count was 15,000 three hours after perforation; her pulse 120, and Temp. 101 F.

Operation three and a half hours after perforation; ether; median incision above navel; as soon as the peritoneum was opened, the contents of the stomach was ejected through the wound, the perforation coming into view at once; this was a round, punched out ulcer about the size of a lead pencil located over the pylorus; the tissues around the ulcer were very much indurated for half an inch or more, and I was compelled to go beyond this area before getting the perforation closed, and this narrowed the pylorus so much that a gastro-enterostomy became necessary. Mayo's posterior no-loop operation was done; one drain was introduced beneath the liver and another into the pelvis through the stab wound. She left the table in good condition, and made an uninterrupted recovery.

She has had better health since this operation than at any time since maturity.

Case 2. Rupture of the gall-bladder; severe hemorrhage from gastric ulcer; posterior gastro-enterostomy; excision of the gall-bladder and appendix; operation six years ago.

The patient a male 58 years of age; was seen the day before operation, and the following history was taken:

He has had some digestive trouble at times for the past twenty years: for the past year has had an attack of acute indigestion on an average of about once a month; has severe pain in the epigastrium lasting from two to three hours, with nausea and vomiting; has never vomited blood; has considerable gas distension with sour stomach and water brash; has lost 45 or 50 pounds in the past year due chiefly to a restricted diet and confinement. No enlargement of the stomach; some tenderness over the pylorus; no tumor; some tenderness over the gall-bladder; slight icteroid color of the skin; conjunctivae slightly yellowed for the past few days, and he has been very much depressed during this time; pulse 60 and rather tense; other organs negative.

While straining at stool, he experienced a sudden, sharp pain in the epigastrium, vomited a considerable amount of clotted blood, became more or less callapsed, and continued to have severe pain in the upper abdomen, and was only partially relieved by a hypodermic containing three-eights of morphia.

I saw him two hours later, and made a diagnosis of perforation of the duodenum, and advised immediate operation. His pulse was 110, and his Temp. 101 F., and White Blood count 14,500. He was removed to the hospital, and operation done four hours after perforation.

Under ether, an incision was made in the mid-line extending down to the navel; some yellowish material was seen to well up into the wound. A careful examination failed to reveal a perforation of the stomach. The gall-bladder was found to be adherent to the transverse colon, and had perforated just at the margins of these adhesions; the gall-bladder was excised and the appendix also which was found to be fibrous; the wound in the colon closed, and a no-loop gastro-enterostomy was done; a wick drain was passed down to the stump of the cystic duct, and there was considerable drainage on the second day. This wick was not removed for about ten days, when it was found to be practically a mass of bile salts, a long gall-stone, and was causing marked irritation and nausea from pressure on the stomach and duodenum. This was replaced by a small rubber tube, but this had to be removed also, and it caused some irritation. All nausea ceased as soon as the drains were removed, and he has made a good recovery, and has been in perfect health since this time.

Case 3. Chronic ulcer near the pylorus; no-loof gastro-enterostomy.

The patient died about two years later of some intercurrent disease.

Mrs. B., age 33; has had digestive trouble for the past ten years; this came after drinking too much water while overheated and caused her to faint, probably causing an acute dilatation; she was somewhat relieved by vomiting. Since this time she has had considerable digestive disturbance; pain after eating which comes on before she leaves the table and usually lasts about two hours; gas distension; palpitation; pain around her heart; gnawing pain in the region of the stomach; recently the pain and soreness in the epigastrium have been constant, and is relieved by a little food; she improves for a while and then gets worse again; has lost about thirty pounds in weight in the past six months; bowels constipated.

Operation under ether; through the right rectus muscle; several scars were found on the first part of the duodenum and one on the pyloric portion of the stomach; a no-loop gastro-enterostomy was done; no anesthetic was given after the clamps were applied until we were ready to close the wound. She came off the table in good condition, and made a perfect recovery; was in a wheel-chair and in the sun-parlor at the end of a week. She went to her home in the country soon after, and I did not see her again.

Case 4. Acute Gastric and Duodenal Dilation; Gastro-Enterostomy.

Operation about six years ago; has remained in good health since.

A female, 22 years of age; has always been in good health until about two months ago when she began to have pain and nausea after eating; some pain in the region of the umbilicus, but most marked in the epigastrium to the left of the mid line; she presses her hand on this point when she walks; appetite poor; has vomited occasionally; pain begins a few minutes after eating and soon becomes more marked; has been troubled with gas distension and acid eructations; is relieved some by taking soda; has had some palpitation and pain around her heart. She has been in bed for four weeks, and has vomited almost daily; has vomited blood; has had tarry stools; very constipated; has vomited incessantly for two weeks almost as soon as any food is ingested; this contains considerable bright blood at times. There is marked pain and tenderness over the left rectus in the epigastric region; she was kept in the hospital for a week on an alkalinse treatment without any improvement, and vomited large quantities of a dark green fluid.

Operation through the right rectus: the duodenum was very much dilated, and there was some dilatation of the stomach; Mayo's no-loop operation was done; she stood the operation well.

On account of the dilated condition of the duodenum, I placed her in bed with the foot elevated; a few days later, this was let down, and she began to vomit again; the foot of the bed was again elevated, and her stomach was washed out daily for three or four days, when all vomiting ceased, and she soon began to take food and make a rapid recovery. She has been seen a number of times in the past few years, and seems to be relieved of her trouble.

Case 5. Mrs. P., age 22 years; has had more or less digestive trouble for three or four years; gas distension, nausea and vomiting; pain after eating; very nervous (neurastheniic); no children. For the past month has been confined to bed most of the time; unable to eat and vomiting almost constantly; some emaciation.

Operation in May, 1908; ether anesthesia; indurated ulcer near the pylorus; Mayo's no-loop operation.

She made an uninterrupted recovery, and has been in perfect health ever since; has gained twenty-five or thirty pounds in weight; has given birth to two children within the past four years.

Case 6. Mrs. P., 26 years of age; the mother of one child; has been under treatment for indigestion for two years or more; considerable gas distension, sour stomach, and pain from one to two hours after meals. Four months ago, she had an attack of gall-bladder colic, and was only relieved by several hypos of morphia, but was tender over the region of the pylorus for about a week; this cleared up, and she seemed quite well and gained some in weight until about a month ago, when she had omre pains and soreness in this region; the soreness did not disappear, and she has scarcely been able to eat anything for a month. There is some nausea.

Operation in April, 1913 under Gas-Oxygen anesthesia. The gall-bladder was drained and found to contain a thick tarry bile but no stones. A thickened, indurated mass wos found to almost occlude the pylorus, and a no-loop gastro-enterostomy was done. Her recovery was as smooth as in the average interval appendix operation, and she was eating almost anything she wanted three or four weeks after operation, and his been in perfect health ever since.

Case 7. Mrs. Z., 57 years of age; has had some digestive trouble for 15 or 20 years; gas distension and sour stomach, heart-burn, and at times has had severe attacks of pain (cramps), with vomiting. Two months ago, she began to have severe pain in the upper abdomen, and noticed a mass about the size of an English walnut in this region; has had several attacks of pain daily, and has been quite tender constantly. When palpating the mass gas can be forced through with a gurgling sound. Has been on a liquid diet for three weeks and has been in bed most of the time. Has been very much nauseated for the past two weeks and has had but very little nourishment. Has had tarry stools at times. The X-Ray picture shows the outlines of a tumor which contracts the pylorus, which appears as a small drawn out tube.

Operation under Gas-Oxygen, October, 1912. When the stomach was drawn out, it was found to contain a large indurated mass, and more than two-thirds of the body of the stomach was also indurated; none of the glands in this region or in the pelvis were enlarged, and we decided to do an excision. The whole of the indurated portion of the stomach was removed, and an anterior gastro-enterostomy was done, as the remaining portion was so small that we were unable to reach the posterior part. She left the table with an increase of only ten beats in her pulse rate and no shock; she vomited a little for 24 hours; she was given cracked ice at once and broth on the second day; she was up in a wheel-chair on the 8th day, and went home at the end of three weeks in better condition than when she came to the hospital. She returned to her home in Florida two months later. She takes food frequently in small quantities but seems to digest normally. Recent reports from her state that her health is better than it has been for several years.

A section was cut from the pylorus and examined by Dr. McLester, who reported it to be carcinoma.

Case 8. W. M. B., male, age 54; referred by Dr. McWhorter, of Riverton, Alabama. Family negative, except that his father was supposed to have died of a malignant tumor of the stomach at 57. General health has always been good up to six or seven years ago, when he began to have some digestive trouble, sour stomach, gas, and acid eructations; has used soda almost constantly. Has been under heavy business strain for the past several years. Digestive trouble has been worse for the past four years. I saw him three years ago, and made a diagnosis of gastric ulcer. Has had a great deal of trouble for three years; pain two or three hours after meals, relieved by taking soda and by vomiting. His condition improves for two or three weeks, and then gets worse; vomits blood during these attacks, and has tarry stools, very much constipated, and this has been worse for four or five months; has lost about fifty pounds in weight.

On admission to the hospital, and very much emaciated, he was put on a diet for about a week, but did not improve, and operation was decided upon.

Under Gas-Oxygen, an incision was made through the upper part of the rectus; a large indurated ulcer the size of a silver dollar was found on the lesser curviture encroaching upon the pylorus; the surrounding tissues were much thickened and indurated, and some enlarged glands were found around the pylorus. Excision of this part of the stomach was considered, but we decided to do a gastro-enterostomy and do the excision later, if we found it to be necessary (a microscopic examination of the glands was negative).

He made a rapid recovery and did not have any more discomfort. He is reported well, and continues to take on flesh and strength.

Case 9. Male, 42; stomach trouble began fifteen years ago; sour stomach, belching, gas; pain through chest to back; pain about two hours after eating; wakes up about two or three o'clock with pain in his abdomen; spits up his food; has a burning sensation with nausea; constipated. Has lost thirty pounds in past two years. He had a suspicious lesion thirty years ago, and about three or four months ago was given Salvarsan. A bismuth meal was given, and marked contractions of the stomach were noted with the foroscope as is found in ulcer beyond the pylorus; some ptosis of the stomach.

Under ether, the stomach was exposed through the rectus muscle, and an ulcer and an indurated mass were found just beyond the pyloric ring. A posterior no-loop gastro-enterostomy was done, and then the pylorus was excised.

He made a smooth recovery, and has been able to take and digest his food well, has gained some flesh, and seems to be relieved of all his old symptoms.

